# Serum EBV antibodies and LMP-1 in Polish patients with oropharyngeal and laryngeal cancer

**DOI:** 10.1186/s13027-017-0141-x

**Published:** 2017-05-30

**Authors:** Sylwia Fołtyn, Małgorzata Strycharz-Dudziak, Bartłomiej Drop, Anastazja Boguszewska, Małgorzata Polz-Dacewicz

**Affiliations:** 1 Department of Virology, Medical University of Lublin, Lublin, Poland; 2 Chair and Department of Conservative Dentistry with Endodontics, Medical University of Lublin, Lublin, Poland; 3 Chair and Department of Public Health, Medical University of Lublin, Lublin, Poland

**Keywords:** EBV antibodies, LMP1, Oropharyngeal cancer, Laryngeal cancer

## Abstract

**Background:**

The association between Epstein-Barr virus (EBV) and the development of head and neck cancer was reported by many researchers. The aim of the present study was to detect EBV DNA and EBV antibodies in 110 Polish patients with oropharyngeal and laryngeal cancer compared to 40 healthy individuals.

**Methods:**

Frozen tumor tissue fragments were tested using nested PCR assay for EBV DNA detection. Sera from all individuals were investigated using ELISA tests to detect the presence of VCA IgM and IgG, EBNA IgG, EA IgG.

**Results:**

EBV DNA was detected in 52.7% of the patients (25% in controls). EBVCA were detected in 94.5%, EBNA in 96.4% and EA in 94.5% of patients. The significantly higher level of EA in the patients suggests EBV reactivation. The majority of patients (83%) were infected with wild-type EBV.

**Conclusion:**

Our study showed that this variant seems to be associated with oropharyngeal and laryngeal cancer in the Polish population.

## Background

The Epstein-Barr virus (EBV) is a ubiquitous gammaherpesvirus that infects more than 90% of the global adult human population [1–3]. For the past two decades, increasing interest has been focused on the EBV-associated cancers including Burkitt’s lymphoma (BL), Hodgkin lymphoma (HL), nasopharyngeal carcinoma and gastric cancer [4–8].

The global burden of mortality from EBV-related cancers accounts for 1.8% of all cancer deaths in 2010 [9]. The trends indicate that both this burden and life-expectancy in the world population will increase. About 92% of all EBV-associated cancer deaths are caused by nasopharyngeal cancer and gastric cancer. After primary infection, EBV establishes latent infection in B lymphocytes with periodic reactivation and viral transmission in the oropharyngeal epithelium. Chronic viral infection is an important risk factor, particularly for tongue cancer and oropharyngeal cancer [1, 7, 8, 10–14]. The association between the latent EBV infection and the development of head and neck cancer (HNC) was reported by several investigators [11, 12, 14, 15].

The present study analysed the serum level of EBV antibodies (VCA IgM and IgG, EBNA IgG, EA IgG) and determined LMP1 variant in patients with oropharyngeal and laryngeal cancers in the Polish population.

## Methods

### Patients

The present study comprised of a group of 110 patients with a diagnosed cancer of the pharynx or larynx who were hospitalized in Otolaryngology Division of the Hospital in Radom, Poland. The results were compared to the control group, involving 40 persons hospitalized at the Otolaryngology Ward due to diseases other than cancer. There were no statistically significant differences between the patients and the control group (age, sex, tobacco and alcohol consumption (Table 1)). The socio-demographic and clinicopathological characteristics of the study group are shown in Table 2 in relation to EBV DNA. The research material consisted of the sera and frozen tumor tissue fragments. This research was approved by the Ethics Committee and is in accordance with the GCP regulations (no. KE-0254/133/2013).

**Table 1:** Epidemiological characteristics of patients and control group

		Patients (*n* = 110)		Controls (*n* = 40)		p
		n	%	n	%	
Sex	female	8	7.3	2	5.0	>0.05
	male	102	92.7	38	95.0	
Age	<49	12	10.9	6	15.0	>0.05
	50–59	46	41.8	16	40.0	
	>60	52	47.3	18	45.0	
Place of residence	urban	72	65.5	28	70.0	>0.05
	rural	38	34.5	12	30.0	
Smoking	yes	94	85.5	36	90.0	>0.05
	no	16	14.5	4	10.0	
Alcohol abuse	yes	50	45.5	14	35.0	>0.05
	no	60	54.5	13	65.0	
EBV DNA	positive	58	52.7	10	25.0	<0.001
	negative	52	47.3	30	75.0

**Table 2:** Epidemiological and clinical characteristics of patients in relation to EBV DNA

		EBV DNA+		EBV DNA-		p
		n	%	n	%	
Sex	female	0	0	8	100	0.0282^*^
	male	58	56.9	44	43.1	
Age	<49	8	50.0	4	50.0	0.8969
	50–59	22	47.8	24	52.2	
	>60	28	50.0	24	50.0	
Place of residence	urban	40	55.6	32	44.4	0.5630
	rural	18	47.4	20	52.6	
Smoking	yes	50	53.2	44	46.8	0.8672
	no	8	50.0	8	50.0	
Alcohol abuse	yes	28	56.0	22	44.0	0.6571
	no	30	50.0	30	50.0	
Histological grading	G1	22	61.1	14	38.9	0.6852
	G2	34	48.57	36	51.43	
	G3	2	50.0	2	50.0	
T stage	T1	6	42.9	8	57.1	0.1826
	T2	28	58.3	20	41.7	
	T3	16	72.7	6	27.3	
	T4	8	30.8	18	69.2	
N stage	N0	30	51.7	28	48.3	0.9768
	N1	10	50.0	10	50.0	
	N2	14	58.3	10	41.7	
	N3	4	50.0	4	50.0	
M stage	M0	58	52.7	52	47.3	
	M1	0	0	0	0	
Location of cancer	pharynx	23	50.0	23	50.0	0.6452
	larynx	35	54.7	29	45.3	
EBV type	wild	48	82.8	-	-	-
	del-LMP1	10	17.2	-	-	

^*^statistically significant

### DNA extraction from fresh frozen tumour tissue

Fragments of the fresh frozen tumour tissue (20 mg), both from the patients with OSCC and from the control subjects (biopsies), were cut and homogenized in a manual homogenizer Omni TH/Omni International/Kennesewa/Georgia/USA. DNA was extracted using a protocol as described in the DNeasy Tissue Kit Handbook (QiagenGmBH, Hilden, Germany). Purified DNA was quantified by spectrophoto-metery (Epoch Microplate Spectrophotometer, BioTek Instruments Inc., Vinooski, Vermont, USA). The isolates were kept at −20 °C until the test was conducted. To verify the quality of the obtained DNA (presence of inhibitors of Polymerase Chain Reaction), a β-globinassay was performed.

### Detection of viruses

EBV DNA detection: All PCR reactions were carried out in the final volume of 25 μl using HotStartTaq DNA Polymerase (Qiagen, Germany). Concentrations of PCR reaction components were prepared as follows: 2.0 mM MgCl_2_, 0.2 mM dNTPs, 0.5 μM of each forward and reverse primers and 0.5 U of HotStartTaq polymerase. During each run the samples were tested together with one negative (nuclease-free water) and positive control (EBV-positive cell line, Namalwa, ATCC-CRL-1432).

### Amplification of EBNA-2 gene

The nested PCR was carried out for amplification of EBNA-2. The sequence of primers used for PCR was as follows: outer pair 5′ – TTT CAC CAA TAC ATG ACC C – 3′, 5′ – TGG CAA AGT GCT GAG AGC AA – 3′ and inner pair 5′ – CAA TAC ATG AAC CRG AGT CC – 3′, 5′ – AAG TGC TGA GAG CAA GGC MC – 3′. 2 μl of extracted DNA was subjected to the PCR mixture with the concentration as described above. The first-round amplification consisted of the activation of polymerase 95 °C for 15 min, 35 cycles of 94 °C for 1 min, 55 °C for 1 min, 72 °C for 2 min and the final extension at 72 °C for 5 min. The second-round amplification was performed with 1 μl of first round PCR product in 30 cycles with an annealing temperature at 60 °C. The amplicons 368 bp, 473 bp in length (depending on the EBV type EBV-1 and EBV-2, respectively) were separated on 2% agarose gel and purified using a Gel-Out kit (A&A Biotechnology, Poland) for further analysis. Purified PCR products were sent to Genomed Warsaw company for sequencing.

### Genotyping of LMP1

PCR screening for the EBV LMP1 subtype based on exon 3, defined as wild-type (wtLMP1) or del-LMP1, was done using specific primers: forward 5′-AGC GAC TCT GCT GGA AAT GAT- 3′; revers 5′-TGA TTA GCT AAG GCA TTC CCA- 3′. Concentrations of PCR reaction components were prepared as follows: 2.0 mM MgCl_2_, 0.2 mM dNTPs, 0.5 μM of each forward and revers primers and 0.5 U Hot Start DNA polymerase and 5 μl of extracted DNA. The reaction mixture (25 μl) was incubated at 95 °C for 15 min., followed by 45 cycles at 94 °C for 1 min., 57 °C for 1 min., 72 °C for 1 min., a final extension at 72 °C for 10 mins. The PCR products were analyzed by gel electrophoresis in a 3% agarose gel and visualized under UV light.

### Serological tests

To detect antibody levels serological tests were used with ELISA method. Designed antibodies: anti-VCA IgM (NovaLisa Epstein-Barr Virus VCA IgM/Nova Tec Immunodiagnostica GmbH/Germany/catalog number: EBVM0150), anti-VCA IgG (NovaLisa Epstein-Barr Virus VCA IgG/Nova Tec Immunodiagnostica GmbH/Germany/catalog number: EBVG0150), and anti-EBNA IgG (NovaLisa Epstein-Barr Virus EBNA IgG/Nova Tec Immunodiagnostica GmbH/Germany/catalog number: EBVG0580), antibodies anti-EA IgG (ELISA-VIDITEST anti-EA (D) EBV IgG/Vidia/Czech Republic/catalog number: ODZ-006). All tests were performed according to the manufacturer’s instructions.

The NovaTec Epstein-Barr Virus (EBV) IgG-ELISA is intended for the qualitative determination of IgG class antibodies against Epstein-Barr virus. Samples are considered positive if the absorbance value is higher than 10% over the cut-off. The level of antibodies is expressed as NovaTec-Units = NTU.

ELISA-VIDITEST anti-EA is semiquantitative test. Samples with absorbances higher than 110% of the cut-off value are considered positive.

### Statistical analysis

Descriptive statistics were used to characterize patient baseline characteristics. The Mann-Whitney *U*-test and Kruskal-Wallis test were used to compare antibody levels. Pearson’s chi-square test was used to investigate the relationship between EBNA2 subtypes and LMP1 subtype and clinical and demographical parameters. Statistical significance was defined as *p* < 0.05.

## Results

EBV DNA was detected in 52.70% of the patients with pharyngeal and laryngeal cancer and in 25.0% of controls (*p* < 0.001). In all patients with EBV DNA type 1 of the virus was detected. Epidemiological and demographical characteristics of the patients and controls are shown in Table 1.

The prevalence of EBV in patients group was significantly higher in males than in females (*p* < 0.05). No statistically significant differences were found between EBV infection and other demographical features, smoking and alcohol consumption (Table 2). The majority of patients (82.8%) were infected with wild type of EBV.

All the patients and controls were EBVCA IgM antibodies negative. IgG EBVCA antibodies were detected in 94.5% of patients, EBNA in 96.4% (Table 3). A statistically significant difference was observed in the prevalence of IgG EA antibodies in patients and controls (94.5% vs 22.5%). High level of EA was stated in 70% of cancer cases and in 0% of controls (*p* < 0.05). The level of IgG EBVCA and IgG EBNA antibodies was higher in the patients than in control group; however, the difference was not statistically significant.

**Table 3:** EBVCA, EBNA and EA level in serum of patients and control group

Antibodies level (IgG)		Patients		Controls		p
		n	%	n	%	
EBVCA	negative	6	5.5	0	0	>0.05
	low	24	21.8	14	35.0	
	high	80	72.7	26	65.0	
EBNA	negative	4	3.6	0	0	>0.05
	low	23	20.9	12	30.0	
	high	83	75.5	28	70.0	
EA	negative	6	5.5	31	77.5	<0.05^*^
	low	27	24.5	9	22.5	
	high	77	70.0	0	0	

^*^statistically significant

Patients with an advanced stage of tumour development (T3-T4 stage) had a significantly higher level of EBNA antibodies than patients with T1-T2 stage (*p* < 0.05), while the level of EBVCA antibodies was the highest in patients with N1 stage. The level of EBVCA antibodies decreased with more advanced nodal stage (*p* < 0.05) (Table 4).

**Table 4:** Association between EBVCA, EBNA, EA antibodies level in patients serum and selected features (*p* value)

Histological grading TN classification	EBVCA IgG	EBNA IgG	EA IgG
Histological grade G	0.9139	0.2222	0.5587
T stage	0.6359	0.0174^a^	0.1759
N stage	0.0126^a^	0.5920	0.4201
EBV type *LMP1* vs *del-LMP1*	0.0379^a^	0.4479	0.3622
Location of cancer	0.8875	0.9363	0.9485

^a^statistically significant

In patients infected with the wild-type of EBV, the level of anti-EBVCA was significantly higher than in cases with EBV type with deletion (*p* < 0.05). There was no relationship between EBV type on the basis of the sequence in LMP1 gene, histological grading, and TN stage (Table 5).

**Table 5:** Relationship between EBV type according to the LMP1 gene sequence and histological grading, TN stage

Histological grading TN classification	EBV *LMP1*		EBV *del-LMP1*		p
	n	%	n	%	
G1	16	72.7	6	27.3	0.05113
G2	30	88.2	4	11.8	
G3	2	100.0	0	0	
T1	6	100.0	0	0	0.2786
T2	24	85.7	4	14.3	
T3	14	87.5	2	12.5	
T4	4	50.0	2	50.0	
N0	26	86.7	4	13.3	0.6293
N1	8	80.0	2	20.0	
N2	12	85.7	2	14.3	
N3	2	50.0	2	50.0	

## Discussion

Two major types of EBV (EBV-1 and EBV-2) have been identified (a classification based on the EBNA2 gene sequence) and according to the researchers they differ in geographic distribution [6, 16]. According to some investigators, these genetic differences in EBV DNA sequence may be responsible for varied interactions with host cells, immunological response and cancer transformation, but the role of specific EBV types in the etiology of different cancers is not fully understood [17, 18]. EBV type 1 has a greater potential to transform B lymphocytes than EBV type 2 and is more common in Western and Asian countries, while EBV-2 is more frequently detected in Africa [16]. The results of our present and previous studies are consistent with the existing reports as type 1 EBV was detected in all examined patients [19]. Similar results were presented by Neves et al. [18], who demonstrated that 96.3% of the examined Portuguese individuals carried EBV type 1.

As the disease progresses, EBV is activated into the replicative stage, in which major viral proteins are expressed [20, 21]. Patients with nasopharyngeal carcinoma show an elevated level of antibodies to several EBV antigens, including the viral capsid antigen (VCA), early antigen (EA) and EB nuclear antigen (EBNA) [22–25]. Traditional assays of EBV antibodies have been very useful in clinical diagnosis of nasopharyngeal cancer (NPC) [26].

Our study revealed that more than half of the patients were EBV positive but also the level of IgG EBV antibodies was higher in patients than in controls and it increased with the tumour development stage. Moreover, the difference in EBV DNA prevalence between patients and controls was statistically significant.

Serological testing was performed both in the patients and in the controls to clarify whether there is a relationship between pharyngeal and laryngeal cancer development and past EBV infection. Various studies revealed that IgG EBV antibodies were detected in a greater part of the examined patients and their level was higher in the patients with pharyngeal and laryngeal cancers than in controls [24, 27, 28]. Other researchers report IgG EBVCA antibodies detection in more than 60% of patients with laryngeal and pharyngeal cancers [29].

Our study did not reveal any significant difference between the presence and the level of EBV antibodies and demographical features of the patients, cancer location and histological grading of malignant lesions. However, patients with an advanced stage of tumour development (T3-T4 stage) had a significantly higher level of EBNA antibodies than patients with T1-T2 stage, while the level of EBVCA antibodies was the highest in patients with N1 stage. The more advanced nodal stage was, the more the level of these antibodies decreased.

The level of EA antibodies among our patients was higher than in control group. Our results are similar to other researchers’ findings and may indicate a reactivation of latent EBV infection, because a high titer of EA antibodies can be seen in cases of reactivation of latent infection [25].

Statistically significant differences were observed by Chen et al. [30] in the scores/levels of six markers (EVB-IgG, VCA-IgG, EA-D p43-IgG, EA-IgG, EA-IgG + EBNA1-IgG, and EA-D p45-IgG) between NPC patients and healthy subjects. The lytic cycle of EBV is important and plays more active roles in oncogenesis [31].

Many authors show that EBV DNA load and IgA antibodies are more effective and useful in the clinical diagnosis and screening of NPC [32–34]. In our study only IgG antibodies were analysed. Immunoglobulins against viral proteins, including EA-IgG, VCA-IgA, and Rta-IgG, may be used as molecular biomarkers for predicting the prognosis of nasopharyngeal cancer. According to Tay et al. [24], EBV DNA load correlated with EA IgA serology titers may be useful in detecting early NPC in screening studies.

EBV is transmitted via oral route and primary infection establishes a lifelong virus latent infection. The establishment of latent EBV infection in premalignant nasopharyngeal epithelial cells and the expression of latent viral genes are crucial features of NPC [14, 35].

Latent infection was divided into different subgroups due to specific viral proteins expression [12, 31, 34]. Nasopharyngeal carcinoma can display both latency type I and II EBV infections [36]. During type I latency EBNA1, EBER1 and 2, BamHI-A rightward transcripts (BART) are expressed, but type II latency can also express latent membrane protein 1 (*LMP1*). The oncogenic role of *LMP1* is well established. In nasopharyngeal carcinoma *LMP1* expression is associated with TNM stage and lymph node metastasis [37]. The EBV variant with a 30 bp deletion ((amino acids 346–355) includes part of C terminal activating region 2) isolated from an NPC tumor had a greater transforming activity than the reference *LMP1* [38]. The 30 bp deletion variant (*del-LMP1*) was first detected in EBV isolated from cell lines derived from NPC patients from Southern China [39]. Molecular studies demonstrated that a higher frequency of nasopharyngeal cancer detected in Asian population contains a variant of EBV *LMP1* gene with a 30-bp deletion (*del-LMP1*) [16, 34].

According to some researchers, EBV *del-LMP1* plays a key role in nasopharyngeal cancer development and might be detected at higher frequencies in NPC patients than in the general population. Other investigators, however, suggest that *del-LMP1* is only a geographic variation – it is more common in the Chinese population but not involved in the pathogenesis of NPC, as no association was found between the *del-LMP1* and NPC. Hadhri et al. [40] found that *del-LMP1* variant was significantly more frequent in NPC (71.42%) than in control biopsies (52%) in Tunisia. Tiwawech et al. [16] also reported that a significant association between the *del-LMP1* variant and NPC susceptibility was found in the Thai. Moreover, the frequency of *del-LMP1* in NPC patients was associated with the clinical stage of NPC [39].

Our study demonstrated that in the Polish population with oropharyngeal and laryngeal cancer *wild-type LMP1* was more frequent (83%). A limitation of our research is, however, small size of *del-LMP1* group, which makes statistical data comparing relationship between EBV type on the basis of the sequence in LMP1 gene and histological grading or TN stage not sufficiently strong. Neves et al. [18] demonstrated that EBV-2 and *wt-LMP1* were associated with NPC in the Portuguese population. Their research performed in a similar ethnic group – Portuguese individuals – revealed no predominance of a specific *LPM1* variant as not only both variants but also co-infection was common in this population. However, contrary to the Chinese population these authors found that the majority of NPC patients were *wt-LMP1*, which pointed to a differential geographic association of EBV-strains with NPC development.

Although the association between EBV infection and head and neck cancer was reported in various studies, the mechanism of malignancy development is still not clear. Understanding the role of the EBV latent genes expressed in pharyngeal and laryngeal cancers is crucial in determining the role of viral infection in the development and progression of cancer in this area.

## Conclusions

Our results reveal that EBV DNA and a high level of antibodies, particularly EA, are most frequent and the wild type EBV is predominant in Polish patients with both pharyngeal and laryngeal carcinoma. However, further studies are necessary to clarify the role of Epstein-Barr virus in cancer development because genetic and epigenetic changes occur after EBV infection.

## Abbreviations

BL: Burkitt’s lymphoma
del-LMP1: deletion variant of latent membrane protein 1
EA: Early antigen
EBNA: Epstein-Barr nuclear antigen
EBV: Epstein-Barr virus
EBVCA: Epstein-Barr viral capsid antigen
HL: Hodgkin lymphoma
HNC: Head and neck cancer
LMP1: Latent membrane protein 1
NPC: Nasopharyngeal cancer
TN: Tumour, node
VCA: Viral capsid antigen
wt-LMP1: wild type latent membrane protein 1
